# Supplementary material for: Widespread release of translational repression across Plasmodium’s host-to-vector transmission event
Source: PLoS Pathog. 2025 Jan 8;21(1):e1012823. doi: 10.1371/journal.ppat.1012823 (PMC11750109; doi:10.1371/journal.ppat.1012823)
Supplement: S1 Fig — (Top) A genomic locus schematic of the unedited wild-type locus and the transgenic locus are shown with anticipated PCR fragment sizes listed. (Bottom) Genotyping PCR results for wild-type (Py17XNL), transgenic parasites (TG), no template controls (NTC), and a positive plasmid control (the plasmid used to create the transgenic line) are shown. (PDF) [file ppat.1012823.s001.pdf]

Rios *et al.* (2024) S1 Fig

A.

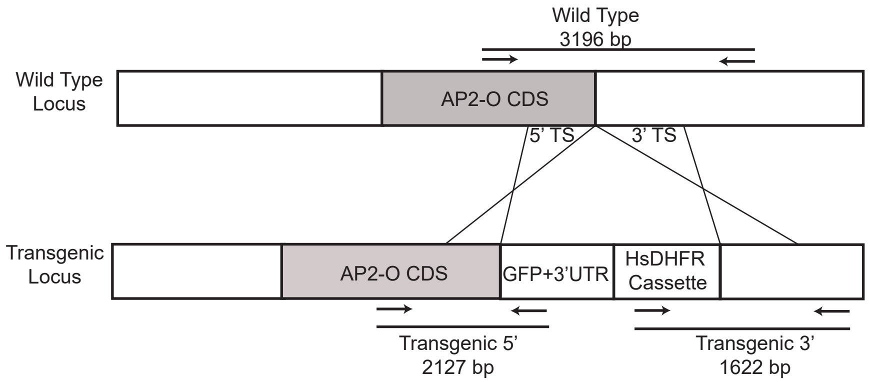

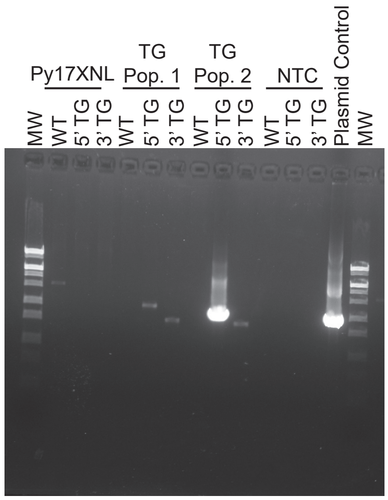

Py1534 - AP2-O::GFP

S1 Fig: Genomic locus and genotyping PCR for the PyApiAP2-O::GFP lines.
(Top) A genomic locus schematic of the unedited wild-type locus and the transgenic locus are shown with anticipated PCR fragment sizes listed. (Bottom) Genotyping PCR results for wild-type (Py17XNL), transgenic parasites (TG), no template controls (NTC), and a positive plasmid control (the plasmid used to create the transgenic line) are shown.
