# Supplementary material for: Widespread release of translational repression across Plasmodium’s host-to-vector transmission event
Source: PLoS Pathog. 2025 Jan 8;21(1):e1012823. doi: 10.1371/journal.ppat.1012823 (PMC11750109; doi:10.1371/journal.ppat.1012823)
Supplement: S2 Fig — (A) Uninfected red blood cells were used as a negative control for the collection of female gametocytes expressing green fluorescent protein (GFP) from a female-enriched promoter. (B) Parasites expressing GFP from the female-enriched pylap4promoter were selected by fluorescence activated cell sorting and collected into RPMI kept at 37°C to prevent gametogenesis. Sorted parasites were fixed with methanol and stained with Giemsa to further validate the collection of female gametocytes (a representative image is shown). (C) Collected cells were assessed by fluorescence microscopy to ensure female gametocytes were specifically enriched by FACS. (D) Uninfected red blood cells stained with DRAQ5 nuclear stain were used as a negative control for the collection of Pys25-positive zygotes. (E) In vitro cultured zygotes were surface stained with α-Pys25 primary antibody (mouse) and α-mouse Alexa Fluor594 secondary antibody, and DRAQ5 to separate the Pys25-positive zygotes from uninfected red blood cells or other parasite life stages. The cultured zygotes also expressed GFP as a fusion protein with PyApiAP2-O::GFP. (F) Collected zygotes were assessed by live fluorescence to ensure the expected population was enriched by FACS. (PDF) [file ppat.1012823.s002.pdf]

Rios *et al.* (2024) S2 Fig

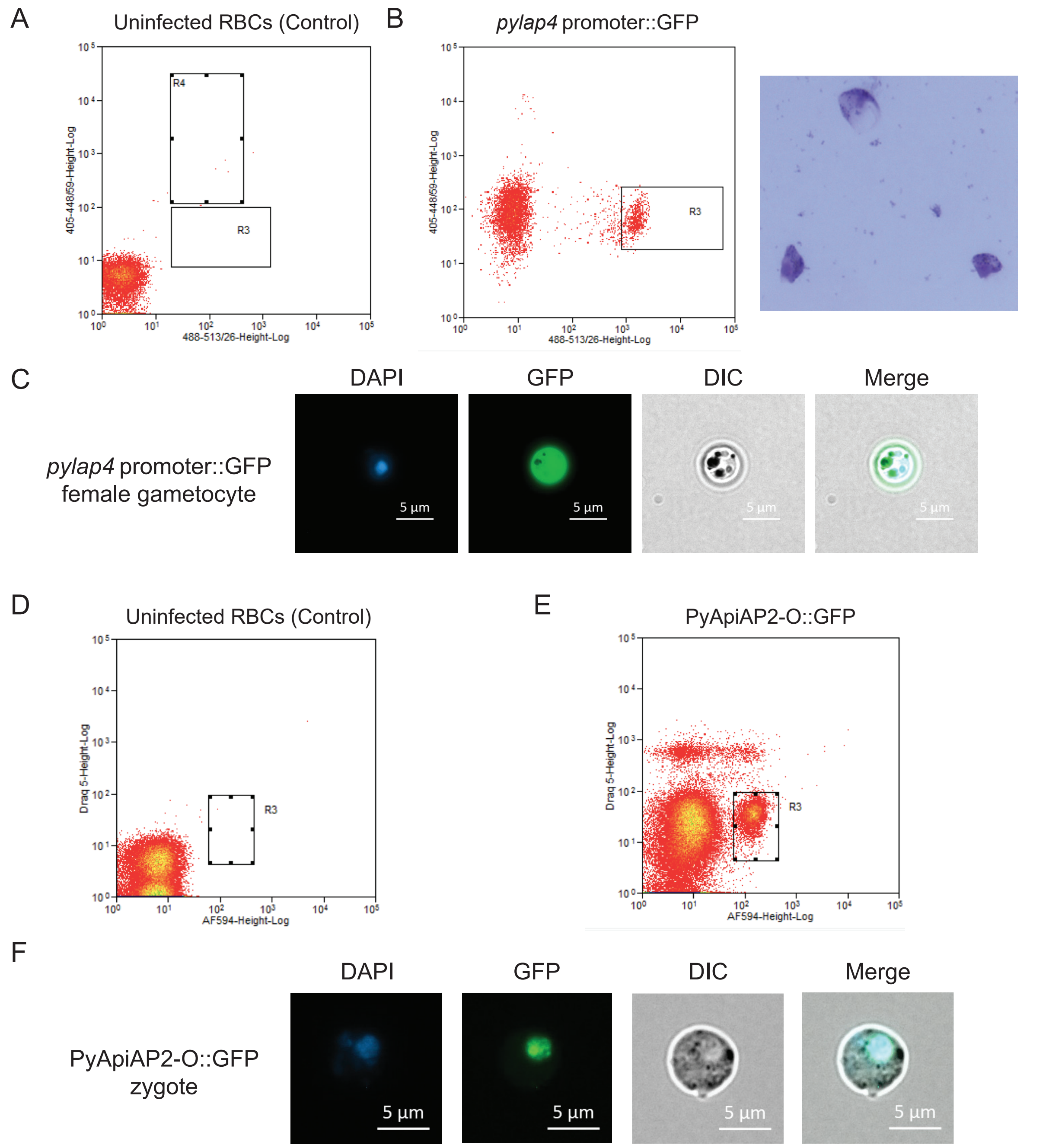

S2 Fig: Flow cytometric enrichment of female gametocytes and zygotes. A. Uninfected red blood cells were used as a negative control for the collection of female gametocytes expressing green fluorescent protein (GFP) from a female-enriched promoter. B. Parasites expressing GFP from the female-enriched *pylap4*promoter were selected by fluorescence activated cell sorting and collected into RPMI kept at 37°C to prevent gametogenesis. Sorted parasites were fixed with methanol and stained with Giemsa to further validate their purity. C. Collected cells were assessed by fluorescence microscopy to ensure female gametocytes were specifically enriched by FACS. D. Uninfected red blood cells stained with DRAQ5 nuclear stain were used as a negative control for the collection of Pys25-positive zygotes. E. *In vitro* cultured zygotes were surface stained with α-Pys25 primary antibody (mouse) and α-mouse Alexa Fluor594 secondary antibody, and DRAQ5 to separate the Pys25-positive zygotes from uninfected red blood cells or other parasite life stages. The cultured zygotes also expressed GFP as a fusion protein with PyApiAP2-O::GFP. F. Collected zygotes were assessed by live fluorescence to ensure the expected population was enriched by FACS.
