# Supplementary material for: Widespread release of translational repression across Plasmodium’s host-to-vector transmission event
Source: PLoS Pathog. 2025 Jan 8;21(1):e1012823. doi: 10.1371/journal.ppat.1012823 (PMC11750109; doi:10.1371/journal.ppat.1012823)
Supplement: S4 Fig — (A) Schematic of TurboID::GFP-tagged PyALBA4, PyDOZI, and an unfused control. Expected protein masses of each fusion protein are provided. (B and C) PyALBA4 was endogenously tagged with GFP (no TurboID), BioID::GFP, or TurboID::GFP (TID). Mixed blood stage parasites were supplemented without (0) or with 150μM biotin for 15 minutes (15’) or 12 hours. Whole-cell lysates were probed with (B) streptavidin-HRP to assess the extent of biotinylation in each sample or (C) α-GFP antibody to confirm that while the efficiency of biotinylation differs between these parasite lines, the tagged proteins are present at qualitatively similar abundances. (D and E) Gametocytes were similarly tested for TurboID activity in an ex vivo culture without biotin supplementation (0) or with 150μM biotin for 15 minutes, or 1, 2, 4, or 12 hours. Unfused TID::GFP gametocytes were compared with (D) PyALBA4::TID::GFP or (E) PyDOZI::TID:GFP. Whole-cell lysates were probed with streptavidin-HRP to assess the extent of biotinylation in each sample. A red asterisk at the bottom of the 1-hour lane indicates that this condition was selected for mass spectrometric analyses. (F) Zygotes were similarly tested for TurboID activity. Zygotes were cultured in vitro for 6 hours, with or without supplementation with 150 μM biotin for the final hour before capture on Pys25-coated magnetic Protein G beads. Unfused TID::GFP zygotes were compared with PyALBA4::TID::GFP or PyDOZI::TID::GFP. Whole-cell lysates were probed with streptavidin-HRP to assess the extent of biotinylation in each sample. (G and H) TurboID-based biotinylated proteins from gametocytes expressing unfused TID::GFP or PyALBA4::TID::GFP were captured on streptavidin-conjugated Dynabeads. The input (“I”), flow-through (“F”), and eluate (“E”) were probed with (F) streptavidin-HRP or (G) α-GFP antibody as above. (I and J) The same experiment but with TID::GFP and PyDOZI::TID::GFP was conducted and probed as in panels F and G. (K) TurboID-based biotiny [file ppat.1012823.s004.pdf]

Rios *et al.* (2024) S4 Fig

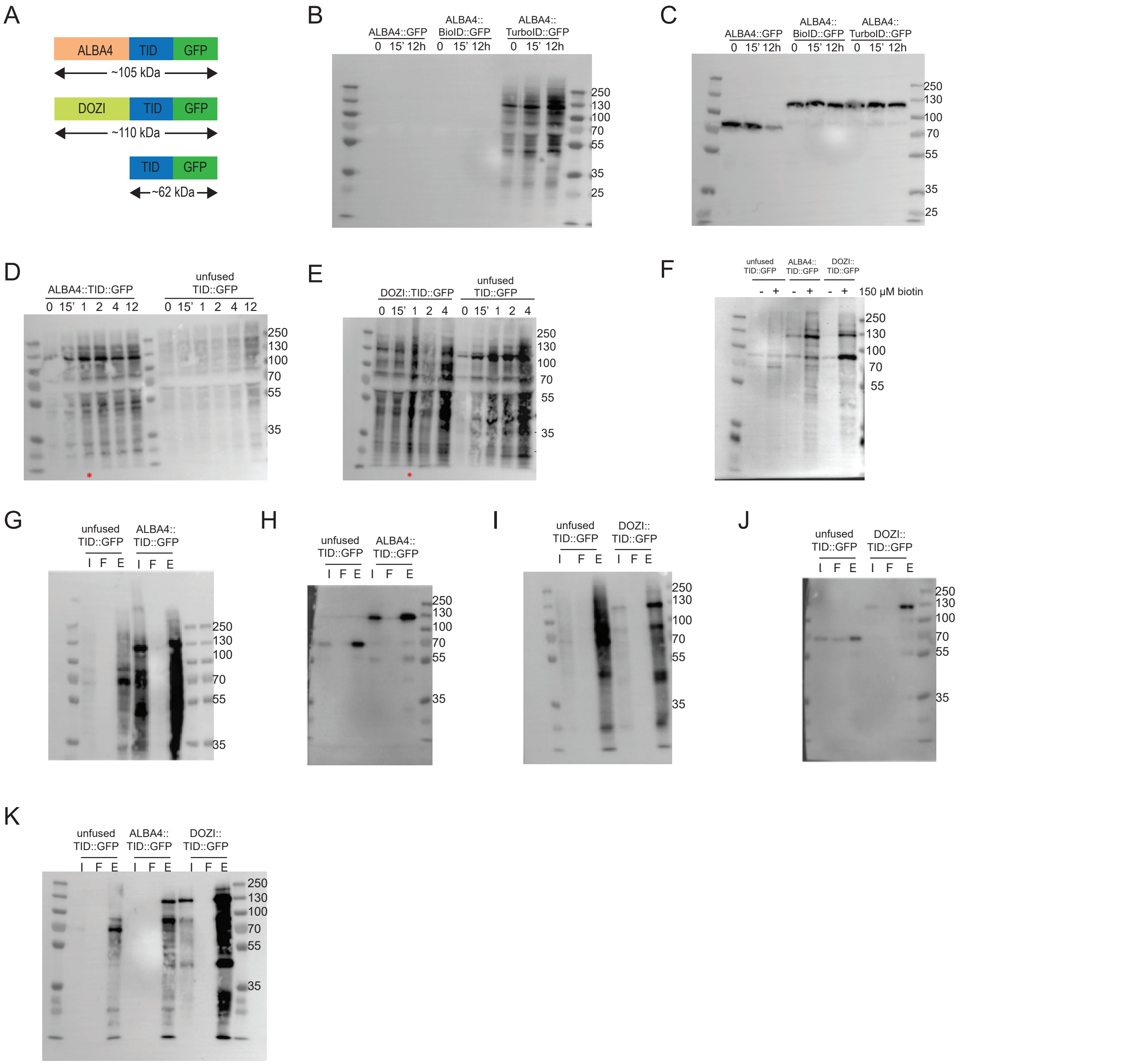

S4 Fig: Quality control blots in support of TurboID experiments with female gametocytes and zygotes. A. Schematic of TurboID::GFP-tagged PyALBA4, PyDOZI, and an unfused control. Expected protein masses of each fusion protein are provided. (B and C) PyALBA4 was endogenously tagged with GFP (no TurboID), BioID::GFP, or TurboID::GFP (TID). Mixed blood stage parasites were supplemented without (0) or with 150uM biotin for 15 minutes (15') or 12 hours. Whole-cell lysates were probed with (B) streptavidin-HRP to assess the extent of biotinylation in each sample or (C) α-GFP antibody to confirm that while the efficiency of biotinylation differs between these parasite lines, the tagged proteins are present at qualitatively similar abundances. (D and E) Gametocytes were similarly tested for TurboID activity in an ex vivo culture without biotin supplementation (0) or with 150uM biotin for 15 minutes, or 1, 2, 4, or 12 hours. Unfused TID::GFP gametocytes were compared with (D) PyALBA4::TID::GFP or (E) PyDOZI::TID:GFP. Whole-cell lysates were probed with streptavidin-HRP to assess the extent of biotinylation in each sample. A red asterisk at the bottom of the 1-hour lane indicates that this condition was selected for mass spectrometric analyses. (F) Zygotes were similarly tested for TurboID activity. Zygotes were cultured in vitro for 6 hours, with or without supplementation with 150 μM biotin for the final hour before capture on Pys25-coated magnetic Protein G beads. Unfused TID::GFP zygotes were compared with PyALBA4::TID::GFP or PyDOZI::TID::GFP. Whole-cell lysates were probed with streptavidin-HRP to assess the extent of biotinylation in each sample. (G and H) TurboID-based biotinylated proteins from gametocytes expressing unfused TID::GFP or PyALBA4::TID::GFP were captured on streptavidin-conjugated Dynabeads. The input ("I"), flow-through ("F"), and eluate ("E") were probed with (F) streptavidin-HRP or (G) α-GFP antibody as above. (I and J) The same experiment but with TID::GFP and PyDOZI::TID::GFP was conducted and probed as in panels F and G. (K) TurboID-based biotinylated proteins from in vitro zygotes expressing unfused TID::GFP, PyALBA4::TID::GFP, or PyDOZI::TID::GFP were captured on streptavidin-conjugated Dynabeads. The input ("I"), flow-through ("F"), and eluate ("E") were probed with (F) streptavidin-HRP.
