# Supplementary material for: Widespread release of translational repression across Plasmodium’s host-to-vector transmission event
Source: PLoS Pathog. 2025 Jan 8;21(1):e1012823. doi: 10.1371/journal.ppat.1012823 (PMC11750109; doi:10.1371/journal.ppat.1012823)
Supplement: S5 Fig — (A) Proteins proximal to PyDOZI::TurboID::GFP and/or PyALBA4::TurboID::GFP in gametocytes were compared to the immunoprecipitated PbDOZI::GFP complex in gametocytes by Mair and colleagues [10]. (B) Proteins proximal to PyALBA4::TurboID::GFP in gametocytes were compared to the immunoprecipitated PyALBA4::GFP complex in gametocytes by Munoz and colleagues [20]. (C) Proteins proximal to PyDOZI::TurboID::GFP in gametocytes were compared to the immunoprecipitated PyCCR4-1::GFP complex in blood stages by Hart and colleagues [87]. (PDF) [file ppat.1012823.s005.pdf]

Rios *et al.* (2024) S5 Fig

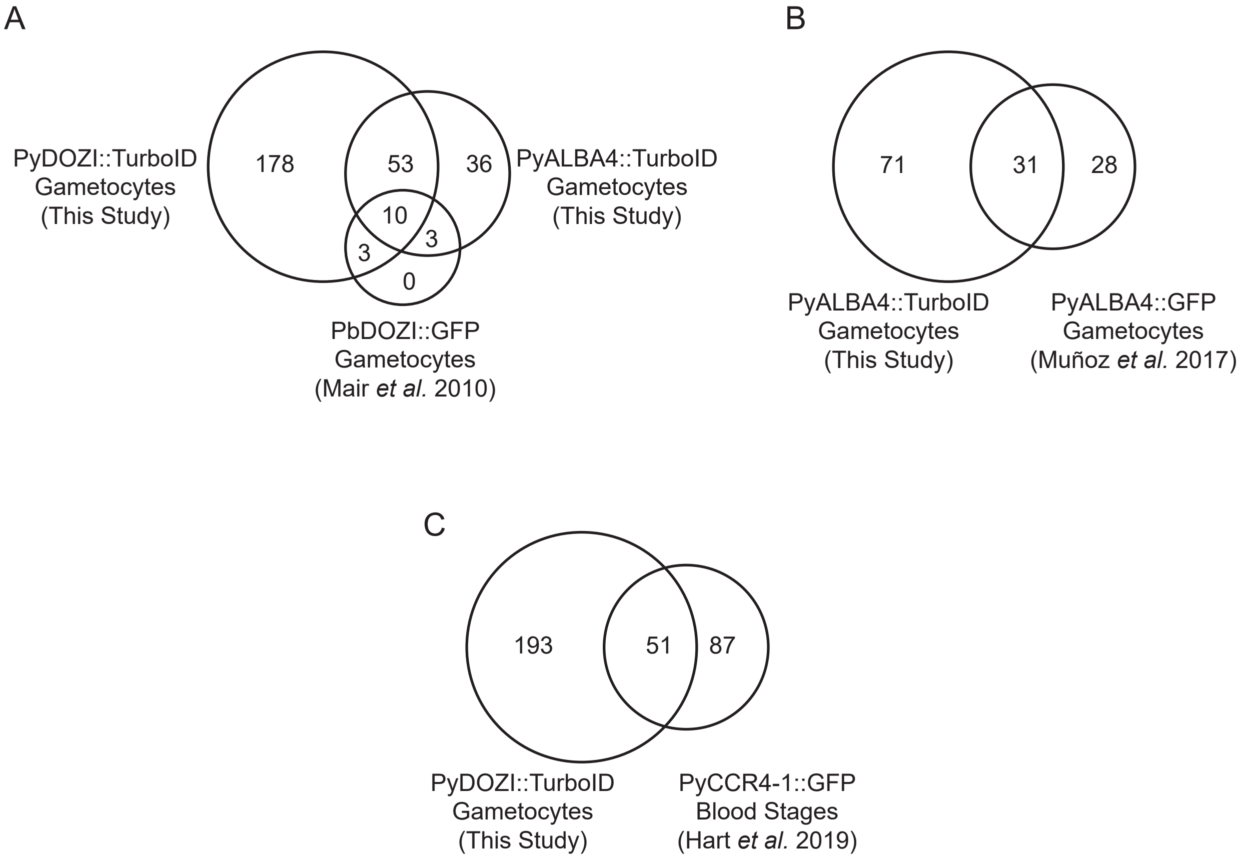

S5 Fig: Comparisons to previously identified protein interactions of DOZI and ALBA4. (A) Proteins proximal to PyDOZI::TurboID::GFP and/or PyALBA4::TurboID::GFP in gametocytes were compared to the immunoprecipitated PbDOZI::GFP complex in gametocytes by Mair and colleagues (10). (B) Proteins proximal to PyALBA4::TurboID::GFP in gametocytes were compared to the immunoprecipitated PyALBA4::GFP complex in gametocytes by Munoz and colleagues (20). (C) Proteins proximal to PyDOZI::TurboID::GFP in gametocytes were compared to the immunoprecipitated PyCCR4-1::GFP complex in blood stages by Hart and colleagues (85).
