# Supplementary material for: Widespread release of translational repression across Plasmodium’s host-to-vector transmission event
Source: PLoS Pathog. 2025 Jan 8;21(1):e1012823. doi: 10.1371/journal.ppat.1012823 (PMC11750109; doi:10.1371/journal.ppat.1012823)
Supplement: S6 Fig — PyDOZI::GFP-expressing (A) or PyNOT-1G::GFP-expressing (B) female gametocytes were used for super-resolution structured illumination microscopy (3D-SIM) imaging to assess protein colocalization. The ImageJ plugin JACoP was used to assess 3D fluorescence colocalization for each pair of proteins indicated [79,106]. The signal intensity from the green channel was compared to the corresponding pixel in the red channel for all Z-stacks of the 3D image to calculate the overlap coefficient. This value varies from 0 to 1, with 0 indicating no fluorescence signal overlap and 1 reflecting complete fluorescence signal colocalization. (PDF) [file ppat.1012823.s006.pdf]

Rios *et al.* (2024) S6 Fig

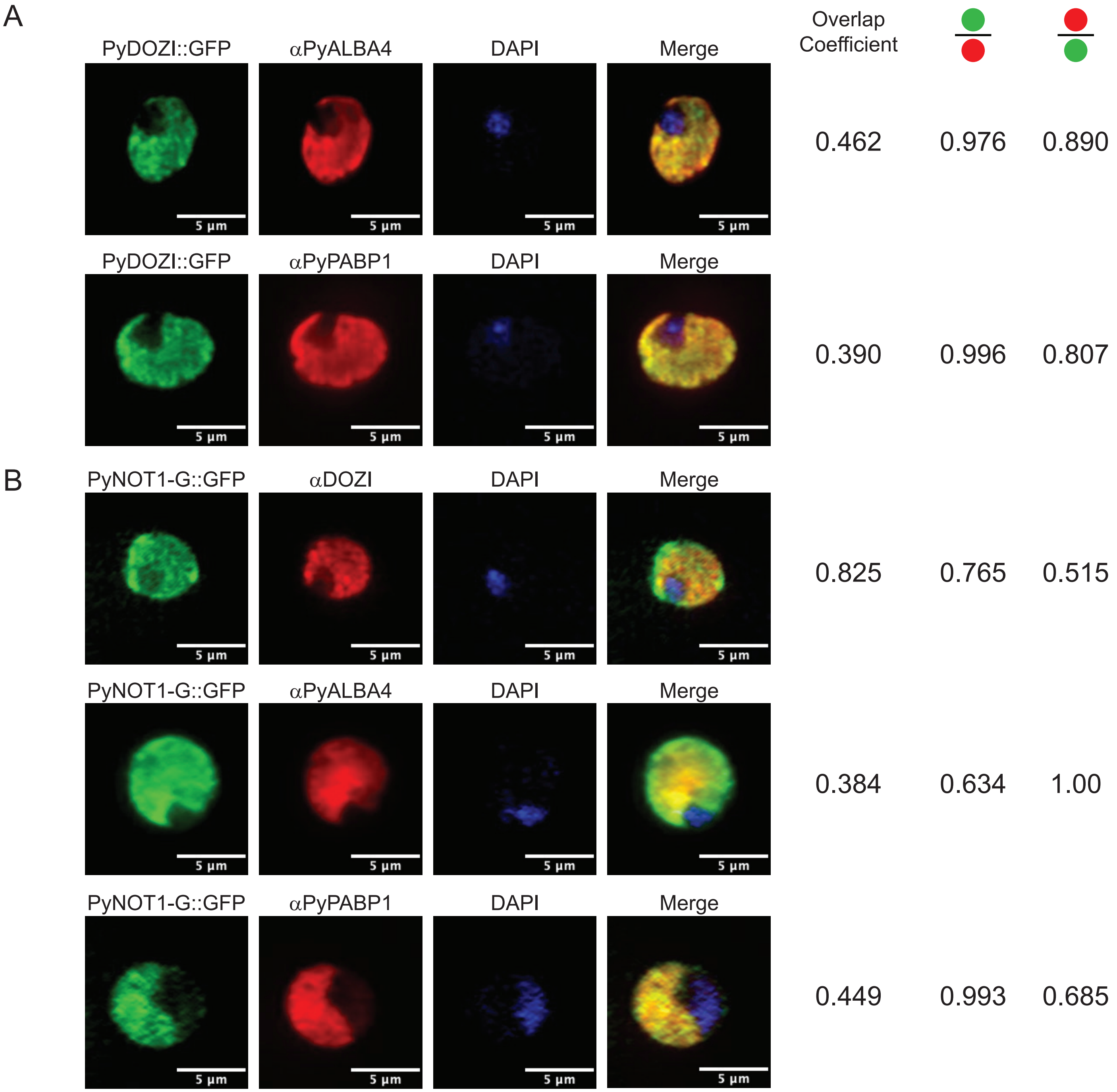

S6 Fig: Colocalization analysis of detected protein-protein interactions in female gametocytes. PyDOZI::GFP-expressing (A) or PyNOT-1G::GFP-expressing (B) female gametocytes were used for super-resolution structured illumination microscopy (3D-SIM) imaging to assess protein colocalization. The ImageJ plugin JACoP was used to assess 3D fluorescence colocalization for each pair of proteins indicated (71, 99). The signal intensity from the green channel was compared to the corresponding pixel in the red channel for all Z-stacks of the 3D image to calculate the overlap coefficient. This value varies from 0 to 1, with 0 indicating no fluorescence signal overlap and 1 reflecting complete fluorescence signal colocalization.
